# Comprehensive versus standard care in post-severe acute kidney injury survivors, a randomized controlled trial

**DOI:** 10.1186/s13054-021-03747-7

**Published:** 2021-08-31

**Authors:** Peerapat Thanapongsatorn, Kamolthip Chaikomon, Nuttha Lumlertgul, Khanitha Yimsangyad, Akarathep Leewongworasingh, Win Kulvichit, Phatadon Sirivongrangson, Sadudee Peerapornratana, Weerachai Chaijamorn, Yingyos Avihingsanon, Nattachai Srisawat

**Affiliations:** 1 Department of Medicine, Central Chest Institute of Thailand, Nonthaburi, Thailand; 2 Excellence Center for Critical Care Nephrology, King Chulalongkorn Memorial Hospital, Bangkok, Thailand; 3 Lerdsin Hospital, Bangkok, Thailand; 4 Critical Care Nephrology Research Unit, Faculty of Medicine, Chulalongkorn University, Bangkok, Thailand; 5 Department of Medicine, Somdech Phra Pinklao Hospital, Bangkok, Thailand; 6 Department of Laboratory Medicine, Faculty of Medicine, Chulalongkorn University, Bangkok, Thailand; 7 Faculty of Pharmacy, Siam University, Bangkok, Thailand; 8 Academy of Science, Royal Society of Thailand, Bangkok, Thailand; 9 Division of Nephrology, Department of Medicine, Faculty of Medicine, King Chulalongkorn Memorial Hospital, Bangkok, 10330 Thailand

**Keywords:** Post-acute kidney injury, Comprehensive care, Severe AKI, Critically ill patients, AKI survivors

## Abstract

**Background:**

Currently, there is a lack of evidence to guide optimal care for acute kidney injury (AKI) survivors. Therefore, post-discharge care by a multidisciplinary care team (MDCT) may improve these outcomes. This study aimed to demonstrate the outcomes of implementing comprehensive care by a MDCT in severe AKI survivors.

**Methods:**

This study was a randomized controlled trial conducted between August 2018 to January 2021. Patients who survived severe AKI stage 2–3 were enrolled and randomized to be followed up with either comprehensive or standard care for 12 months. The comprehensive post-AKI care involved an MDCT (nephrologists, nurses, nutritionists, and pharmacists). The primary outcome was the feasibility outcomes; comprising of the rates of loss to follow up, 3-d dietary record, drug reconciliation, and drug alert rates at 12 months. Secondary outcomes included major adverse kidney events, estimated glomerular filtration rate (eGFR), and the amount of albuminuria at 12 months.

**Results:**

Ninety-eight AKI stage 3 survivors were enrolled and randomized into comprehensive care and standard care groups (49 patients in each group). Compared to the standard care group, the comprehensive care group had significantly better feasibility outcomes; 3-d dietary record, drug reconciliation, and drug alerts (*p* < 0.001). The mean eGFR at 12 months were comparable between the two groups (66.74 vs. 61.12 mL/min/1.73 m^2^, *p* = 0.54). The urine albumin: creatinine ratio (UACR) was significantly lower in the comprehensive care group (36.83 vs. 177.70 mg/g, *p* = 0.036), while the blood pressure control was also better in the comprehensive care group (87.9% vs. 57.5%, *p* = 0.006). There were no differences in the other renal outcomes between the two groups.

**Conclusions:**

Comprehensive care by an MDCT is feasible and could be implemented for severe AKI survivors. MDCT involvement also yields better reduction of the UACR and better blood pressure control.

*Trial registration* Clinicaltrial.gov: NCT04012008 (First registered July 9, 2019).

**Supplementary Information:**

The online version contains supplementary material available at 10.1186/s13054-021-03747-7.

## Introduction

Acute kidney injury (AKI) is estimated to occur in 7–18% of patients in hospital, and approximately 50% of patients admitted to the intensive care unit (ICU) [1, 2]. Moreover, AKI survivors are at an increased risk of chronic kidney disease (CKD), end-stage renal disease, progression of albuminuria, and mortality [3–6]. Post-severe AKI survivors also have a higher risk of heart failure, major atherosclerotic cardiovascular disease events, and all-cause death besides poor renal outcomes [7–10]. These incidents are more severe in resource-limited settings. In Thailand, AKI in patients admitted to the ICU occurred in 2471 of 4688 patients (52.9%), with 28.9% of them classifying as stage 3. Moreover, only 29% of AKI patients had renal recovery at the time of hospital discharge [11].

Many AKI survivors were discharged with different degrees of renal function and renal recovery [12]. Currently, a lack of evidence exists to guide the timing, frequency, and methods to evaluate kidney function and prevent poor outcomes among patients following an episode of severe AKI. In CKD patients, there have been many impressive results from the use of a multidisciplinary care team (MDCT) to improve renal outcomes and mortality [13–17]. Interestingly, AKI survivors with a nephrologists follow-up had an improved all-cause mortality [18]. However, no study so far demonstrated the benefit of the implementation of a MDCT for post-severe AKI survivors. Therefore, this study aims to demonstrate the feasibility and outcomes of implementing comprehensive care in post-severe AKI survivors.

## Materials and methods

### Trial design and oversight

The study was a prospective open-label randomized control trial (RCT) at King Chulalongkorn Memorial Hospital (KCMH) in Thailand from August 2018 to February 2021. The trial was registered at clinicaltrial.gov (NCT04012008) and was approved by the institutional review board at KCMH (IRB No. 005/62). The investigators informed patients or their surrogates concerning the study orally and written informed consent was given before entry into the study.

### Participants

The inclusion criteria were all adult patients (age ≥ 18 y old) who had survived from AKI stage 2–3, as defined by The Kidney Disease: Improving Global Outcomes (KDIGO) 2012 classification [19]. The exclusion criteria were end-stage kidney disease patients who were receiving chronic renal replacement therapy (RRT) or having an estimated glomerular filtration rate (eGFR) of less than 15 mL/min/1.73 m^2^ using the CKD-EPI Creatinine Eq. (2009) prior the admission, kidney transplantation patients, moribund patients whose survival to 1 month was unlikely due to an uncontrollable comorbidity (i.e., end-stage liver or heart disease, untreatable malignancy), and patients who issued the desire not to be included in a follow-up.

### Randomization and intervention

Patients were recruited and randomized at the hospital discharge in a 1:1 ratio (configured by web-based block randomization) and were stratified by their dialysis-dependent status at discharge to receive post-AKI follow-up with either comprehensive or standard care for 12 months. The first visit in each group was within 4 weeks after discharge, depending on the severity of the AKI, dialysis status, and renal recovery status at discharge. Patients with dialysis-requiring AKI or non-recovery AKI were followed up within 1–2 weeks post-discharge, while patients with renal recovery were followed up at 2–4 weeks post-discharge. All patients were then scheduled at the post-AKI clinic every 3 months until the end of the study at 12 months (a total of five visits). To avoid contamination between two groups, we appointed patients for each group on a different date. For patients who were still dialysis dependent or need nephrologist consultation in the standard care group, a different nephrology team from the MDCT group was appointed at the follow up visit. Similarly, to minimize bias, we used the same document format and laboratory protocol in both groups. Every patient visit in both groups had a clinical assessment. A routine laboratory and intervention consisted of a renal function test by serum creatinine and urine albumin: creatinine ratio (UACR), blood chemistry, blood sugar, lipid profile, 24-h urine output analysis to calculate the dietary protein intake and dietary salt intake, blood pressure measurement (BPM), and quality of life (QOL) measurement using the EQ-5D-5L index scores [20]. However, the QOL was only measured at the first visit, and then at the 6- and 12-months follow-up (Additional file 1: Figure S1).

The MDCT who followed up patients in the comprehensive care groups consisted of nephrologists, renal nurses, renal pharmacists, and nutritionists. In the comprehensive care group, we focused on the process of care to improve renal and non-renal outcomes. The responsibility of our MDCT is described in Additional file 1: Table S1. Nephrologists were the principal physicians providing co-ordination with the other MDCT members to manage all transition care post-AKI, such as prevention of CKD progression, preparation of renal replacement therapy (RRT), or conservative therapy. Nephrologists were also responsible for dialysis-related conditions; such as dialysis prescription, vascular access preparation, dry weight adjustment, and medical related conditions (ie: blood pressure control, blood sugar control, anemia management, bone and mineral disease management, volume management, cardiovascular risk management, etc.).

The renal pharmacist took an essential role in documenting the drug reconciliation, which provided details of medications that the patients had been taking and alerted the nephrologists (drug alert) when they found a potential harmful medication; particularly with NSAIDs and herbal medicine. The renal pharmacist also deals with drug conflicts or discrepancies: such as dosing errors, omissions, duplications, drug interactions, or nephrotoxins. This process was documented in the medication reconciliation sheet for every visit, and then scanned to the electronic medical record (EMR). Similarly, drug alerts were also recorded in the EMR. The pharmacists also provided medication education (regarding how some drugs can exacerbate AKI such as nonsteroidal anti-inflammatory agents or antibiotics), pre-operative medication management, and adjustment of the medication dosage based on the renal function.

The renal nutritionists provided individualized dietary and nutritional counseling regarding the intake levels of calories, protein, sodium, potassium, phosphorus, and fluid to prevent CKD progression, dyskalemia, hyperphosphatemia, and hypervolemia based on recent nutrition guidelines [21–23]. For nutritional evaluation, the nutritionist documented the nutrition assessment on every visit using the Nutrition Alert Form (NAF). Dietary compliance was monitored by documenting a 3-d dietary recall, defined as the details of food consumed over the previous 3 d.

Additionally, nurses acted as the coordinator between the patients and the MDCT. Nurses appointed patients to the clinic, encouraged patients to visit the clinic, and contacted patients with the team by phone or the LINE application.

Moreover, our MDCT provided knowledge to patients on every visit with regards to the prevention of CKD progression, dialysis education, lifestyle modification, exercise, smoking cessation, etc. We also emphasized that patients record home (H)BPM, and self-monitor blood glucose. The (H)BPM and glucose measures were then sent directly to our MDCT via LINE group (LINE Application), so the MDCT could adjust medications or recommendations for the patients.

In addition, only internists oversaw patients’ follow-ups in the standard care group. All internists were aware of the study, and they were well educated about post-AKI care; which included medication classes avoidance, and nutritional recommendations. The internists managed the medical condition, including the blood pressure control, blood sugar control, anemia management, bone and mineral disease management, volume management, cardiovascular risk management, as per the nephrologists. However, for patients who required dialysis or impending dialysis, nephrologist consultation was provided by a different team from the comprehensive care group. This team was allowed to manage the dialysis-related conditions. Likewise, the medical conditions mentioned above could be consulted by the nephrologists, based on the decision of the internists. All documents of the internist were recorded in the EMR, including information of the patient’s education, medication use, drug reconciliation, drug alert, or nutrition recommendation by the internist.

### Study measurements

Baseline demographic and biochemical data were collected from the EMR. Baseline creatinine was obtained from the most recent, lowest creatinine level at 7–365 days prior to admission from the EMR. However, for patients without a baseline creatinine or with missing data, we used back-calculation by reversing the MDRD equation using age, sex, and an assumed normal eGFR of 75 mL/min/1.73 m^2^ [24].

Baseline UACR was UACR at 3 months follow-up which was obtained from the EMR. As mentioned above, we used the same document format and laboratory protocol in both groups. All the documentation recorded by both the nephrologist and internists were obtained from the EMR; as well as the medication reconciliation and drug alert notifications from the pharmacists, and dietary records from the nutritionists. The BPM was performed using the mean of three automated BPM at an office visit, where the patient was seated and allowed 5 min of quiet rest before measurement. Anti-hypertensive medications, including the renin–angiotensin–aldosterone-system inhibitor (RAASI), were adjusted based on the decision of the physicians. The QOL was assessed with The Thai Version of the EQ-5D-5L Health Questionnaire by an experienced nurse who was blinded to the study [22, 28].

### Outcomes

The primary outcomes were the feasibility of the process of care by a MDCT. The outcomes were comprised of the four groups of: (i) the rate of loss to follow up; (ii) the rate of 3-d dietary recall, defined as details of food consumed in the past 3 d documented by the nutritionists; (iii) the rate of drug reconciliation, defined as details of medication that the patients had been taking as documented by the pharmacists; and (iv) the rate of drug alerts, defined as any episode of notification to the pharmacists of medication that is potential harmful or may cause interaction. The outcomes of the 3-d dietary recall and drug reconciliation were counted only when there were the documentations of these processes for every visit. Only one episode of drug alert during the period of follow-up was utilized for calculating their outcomes. Patients with loss to follow-up or death before the 12-months visit were not used in the calculation of the primary outcome.

The secondary outcomes were the renal outcomes and non-renal outcomes at 12 months. Five renal secondary outcomes were defined as follows. Firstly, major adverse kidney events (MAKE) in 365 d consisted of death, incident dialysis (a requirement for RRT), and persistent renal dysfunction (doubling of serum creatinine or an eGFR of more than 50% from the baseline) [25]. Secondly, renal recovery, defined as a return of the serum creatinine to baseline or < 1.5 times from baseline and no ongoing need for RRT or currently receiving RRT as acute kidney disease and renal recovery: consensus report of the Acute Disease Quality Initiative (ADQI) 16 Workgroup [12]. Thirdly, incidence of CKD, defined as newly diagnosed CKD by eGFR criteria in patients with no previous history of CKD. Fourthly, the progression of CKD, defined as a change in the stage of CKD by eGFR criteria following the KDIGO 2012 classification [19]. Fifthly, the progression of albuminuria was defined as a change in staging of the albuminuria criteria following the KDIGO 2012 classification from at 3 months to 12 months post-discharge [19]. Lastly, the rate of recurrent AKI, defined as a new episode of AKI following the KDIGO 2012 classification [26].

The non-renal secondary outcomes included the rate of blood pressure control (defined as blood pressure < 140/90 mmHg in non-hypertensive patients and < 130/80 mmHg in previously hypertensive patients, as per the 2018 European Society of Cardiology (ESC) and the European Society of Hypertension (ESH) Guidelines for the management of arterial hypertension) [27], rate of RAASIs usage, rate of rehospitalization (defined as unplanned hospitalization), and the QOL after post-AKI follow up.

### Sample size calculation

As a feasibility study, we aimed to compare the different processes of care between comprehensive care and standard care. At least thirty patients were required to detect a 50% absolute difference in the proportion of feasibility outcome (at least one of the parameters; loss to follow up rate, 3-day dietary recall, drug reconciliation or drug alert) between the comprehensive care group and standard care group with a power of 80% (*β* = 0.2) at a 5% significance level (*α* = 0.05). The trial was stopped on 31^st^ January 2021 after recruiting 98 patients (49 for each group).

### Statistical analyzes

Primary and secondary outcomes were analyzed according to the per-protocol analysis principle, with excluded patients who were lost to follow-up and/or death. Continuous variables are presented as the mean ± one standard deviation (SD) in case of a normal distribution and as a median and interquartile range (IQR) in case of non-normally distributed variables. The student’s t-test or Mann–Whitney test was used to analyze the differences between two continuous variables. Categorical variables were characterized by numbers with percentages and were compared using the Chi-square test or Fisher’s exact test, as appropriate. All statistical analyses were performed with SPSS Version 22 software (SPSS, Chicago, IL), and figures were drawn using GraphPad Prism 8 (GraphPad Software Inc., California, USA).

## Results

### Participants

**Fig. 1:**
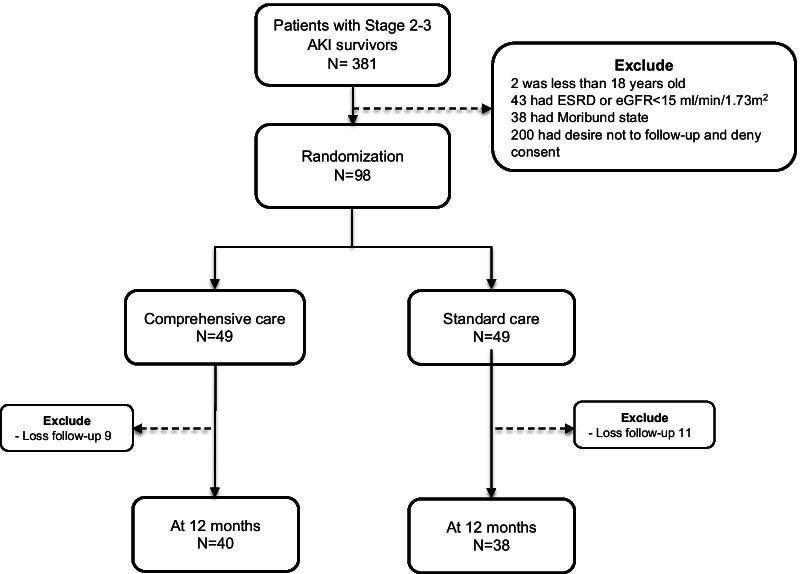
Flow chart of the study. Abbreviations: AKI, acute kidney injury; ESRD, end-stage renal disease

From August 2018 through January 2021, a total of 381 patients from the KCMH met the provisional eligibility requirement. Of these patients, 98 patients were randomized into the comprehensive and standard care groups (49 per group). In the comprehensive care group, nine patients (18.4%) were lost to follow-up and seven (17.1%) died before the end of the 12-month study period. While in the standard care group, there were eleven (22.4%) and five (12.8%) patients who were lost to follow-up and died before the end of follow up, respectively (Fig. 1).

Baseline characteristics were similar between the two groups except that the comprehensive care group had a higher average age than the standard care group (69.7 ± 13.8 y vs. 61.4 ± 16.9 y, *p* = 0.009) (Table 1). The most common AKI causes were sepsis (27.6%) and cardio renal syndrome (25.2%). The RRT rate during admission, RRT dependence at discharge, and renal recovery rate were not significantly different between both groups.

**Table 1:** Demographic, clinical, and biochemical data between comprehensive care and standard care

Parameters	Total (*n* = 98)	Comprehensive care (*n* = 49)	Standard care (*n* = 49)	*p* value
Gender (Male), *n* (%)	53 (54.1%)	27 (55.1%)	26 (53.1%)	0.84
Age, mean (SD)	65.5 (15.9)	69.7 (13.8)	61.4 (16.9)	**0.009**
*Underlying disease*				
Diabetes mellitus, *n* (%)	55 (56.1%)	29 (59.2%)	26 (53.1%)	0.54
Hypertension, *n* (%)	70 (71.4%)	35 (71.4%)	35 (71.4%)	1.00
CKD, *n* (%)	45 (45.9%)	22 (44.9%)	23 (46.9%)	0.84
Liver disease, *n* (%)	14 (14.3%)	6 (12.2%)	8 (16.3%)	0.56
Coronary artery disease, *n* (%)	26 (26.5%)	11 (22.4%)	15 (30.6%)	0.36
Congestive heart failure, *n* (%)	32 (32.7%)	13 (26.5%)	19 (38.8%)	0.20
Cerebrovascular disease, *n* (%)	14 (14.3)	10 (20.4%)	4 (8.2%)	0.08
Malignancy, *n* (%)	19 (19.4%)	10 (20.4%)	9 (18.4%)	0.80
*AKI staging:*				0.51
Stage 2 AKI	6 (12.2%)	4 (8.2%)	6 (12.2%)	
Stage 3 AKI	43 (87.8%)	45 (91.8%)	43 (87.8%)	
*Cause of AKI:*				0.57
Renal hypoperfusion	18 (18.4%)	8 (16.3%)	10 (20.4%)	
Sepsis	27 (27.6%)	15 (30.6%)	12 (24.5%)	
Nephrotoxic	11 (11.2%)	6 (12.2%)	5(10.2%)	
Cardio renal syndrome	25 (25.5%)	9 (18.4%)	16(32.7%)	
Obstructive uropathy	4 (4.1%)	2(4.1%)	2(4.1%)	
Systemic disease	8 (8.2%)	6(12.2%)	2(4.1%)	
Other	5 (5.1%)	3(6.1%)	2(4.1%)	
RRT during admission, *n* (%)	70 (71.4%)	33 (67.3%)	37 (75.5%)	0.37
Baseline serum creatinine (mg/dL), median (IQR)^c^	1.25 (0.88, 2.31)	1.31 (0.89, 2.42)	1.25 (0.80, 2.18)	0.62
Baseline GFR (mL/min/1.73 m^2^), median (IQR)^a^	51.50 (22.39, 87.32)	48.61 (19.86, 82.11)	52.90 (29.18, 87.97)	0.46
Discharge creatinine (mg/dL), median (IQR)	1.87 (1.26, 3.31)	1.53 (1.04, 2.93)	1.71 (1.29, 2.49)	0.66
Discharge GFR (mL/min/1.73 m^2^), median (IQR)^a^	30.80 (17.45, 52.83)	42.14 (18.73, 71.90)	32.06 (21.57, 56.70)	0.84
Baseline urine NGAL at enrollment (ng/mL) median (IQR)	472.85 (193.50, 1686.75)	472.85 (175.68,1481.25)	631.50 (188.50,1874.25)	0.92
Hospital length of stay (d), median (IQR)	16 (11, 31)	15 (11.25,30)	16 (11,34)	0.84
ICU admission, *n* (%)	75 (76.5%)	36 (73.5%)	39 (79.6%)	0.48
ICU length of stay (d), median (IQR)	8 (5,13)	9.5 (5.75,13.5)	8 (4.75,13.25)	0.28
RRT dependence at discharge date, *n* (%)	16 (16.33%)	8 (16.3%)	8 (16.3%)	1.00
Renal recovery at discharge date, *n* (%)^b^	71 (72.45%)	33 (67.3%)	38 (77.6%)	0.37

Significant values are shown in bold type, Data are shown as median (IQR) or mean (SD)

AKI, acute kidney injury; GFR, glomerular filtration rate; ICU, intensive care unit; IQR, interquartile range; NGAL, neutrophil gelatinase-associated lipocalin; RRT, renal replacement therapy; SD, standard deviation

^a^Estimated GFR was calculated by the CKD-EPI creatinine Eq. (2009)

^b^Renal recovery was defined as the serum creatinine level had returned to baseline or < 1.5 times from baseline and not ongoing need for RRT or currently receiving RRT

^c^Baseline serum creatinine was missing for 5 patients (2 in comprehensive care and 3 in standard care)

### Feasibility of comprehensive care

Patients in the comprehensive care group had a significantly higher adherence to the process of post-AKI care (Table 2). However, four patients (two lost to follow-up and two died) in the comprehensive care group and six patients (two lost to follow-up and four died) in the standard care group who were previously RRT-dependent at discharge were excluded. The result of nutrition care by nutritionists using a 3-d dietary recall was significantly different between patients in the comprehensive and standard care groups (100% vs. 0%, respectively, *p* < 0.001), as were the pharmacists’ rate of drug reconciliation and drug alert care (100% vs. 0% and 33.3% vs. 0%, respectively, both *p* < 0.001). However, the follow-up loss rate was not significantly different between the two groups (18.4% and 22.4% in the comprehensive and standard care groups, respectively, *p* = 0.62).

**Table 2:** Feasibility outcomes of the patient

	Comprehensive care	Standard care	*p* value
Loss to follow up rate, *n* (%)	9/40 (18.4%)	11/38 (22.4%)	0.62
Rate of 3-day dietary recall using, *n* (%)^a^	33/33 (100.0%)	0/33 (0%)	**< 0.001**
Rate of drug reconciliation, *n* (%)^a^	33/33 (100.0%)	0/33 (0%)	**< 0.001**
Rate of drug alert, *n* (%)^c^	11/33 (33.3%)	0/33 (0%)	**< 0.001**

Significant values are shown in bold type

^a^Dietary recall; details of food consumed in the past three days documented by the nutritionist

^b^Drug reconciliation; details of medication that the patients had been taking as documented by the pharmacist

^c^Drug alert; any episode of pharmacists' notification of medication that potential harmful or conflict or discrepancy of medication

### Renal outcomes

**Fig. 2:**
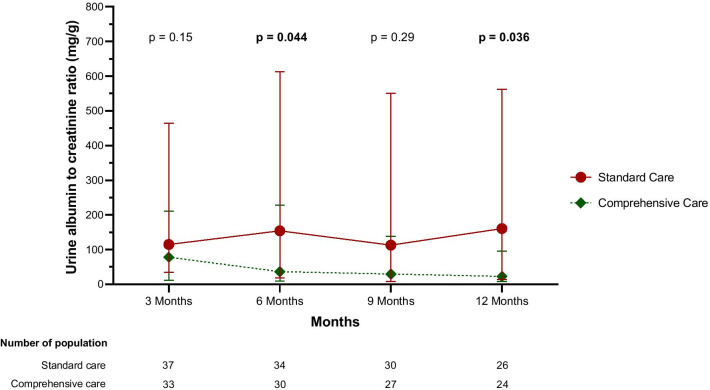
Trend in the median UACR. Data are shown as the median (IQR) and excluded patients with renal replacement therapy and missing data

During follow-up, the comprehensive care group had a significantly reduced UACR compared to the standard group from 3 months onwards, and was lowest at 12 months (36.83 vs. 177.70 mg/g, respectively, *p* = 0.036) (Table 3, Fig. 2, Additional file 1: Table S4). The comprehensive care group also had comparable rates of progression of albuminuria compared to the standard care group (6.1% vs. 18.2%, *p* = 0.26).

**Table 3:** Outcome at 12 months follow up

Parameters	Comprehensive care	Standard care	*p* value
Serum creatinine (mg/dL)*	1.14 (0.80,1.46)	1.05 (0.84,2.08)	0.49
eGFR (mL/min/1.73 m^2^)*	66.74 (30.77)	61.23(35.16)	0.54
UACR (mg/g)*	36.83 (13.39,131.90)	177.70 (47.12,745.71)	**0.036**
MAKE 365, *n* (%)^a^	13/40 (32.5%)	11/38 (28.9%)	0.73
Death, *n* (%)	7/40 (17.1%)	5/38 (12.8%)	0.59
RRT, *n* (%)	6/33 (18.2%)	3/33 (9.1%)	0.28
Doubling serum creatinine, *n* (%)	1/33 (3.0%)	4/33 (12.1%)	0.36
Renal recovery, *n* (%)^b^	8/16 (50%)	4/11 (36.4%)	0.70
New RRT, *n* (%)^c^	1/28 (3.6%)	2/31 (6.5%)	0.62
New CKD, *n* (%)^d^	3/22 (13.6%)	4/19 (21.1%)	0.69
CKD progression, n (%)^e^	7/11 (63.6%)	11/14 (78.6%)	0.41
Progression of albuminuria, n(%)^f^	2/33 (6.1%)	6/33 (18.2%)	0.26
Recurrent AKI, *n* (%)^g^	6/33 (18.2%)	6/33 (18.2%)	1.00
Readmission, *n* (%)^h^	27/49 (55.1%)	24/49 (49%)	0.54
Blood pressure control, *n* (%)^i^	29/33 (87.9%)	19/33 (57.6%)	**0.006**
RAAS inhibitor use, *n* (%)	18/33 (54.5%)	14/33 (42.4%)	0.33
24-h urine sodium (mmol)	112.82 (63.17)	127.69 (54.23)	0.35
24-h dietary protein intake (g/kg/d)	1.01 (0.32)	0.85 (0.28)	0.07
Albumin (g/dL)	4.1 (3.8,4.4)	4.0 (3.8,4.4)	0.59
EQ-5D-5L index scores^j^	0.96 (0.90,1.00)	0.99 (0.80,1.00)	0.80

Significant values are shown in bold type, *Data excluded patients with RRT

AKI, acute kidney injury; CKD, chronic kidney disease; GFR, glomerular filtration rate; RAAS, renin–angiotensin–aldosterone-system; RRT, Renal replacement therapy; SD, standard deviation; UACR, urine albumin creatinine ratio

^a^MAKE 365; major adverse kidney events at 365 d were comprised of death, incident dialysis (requirement for CRRT), and persistent renal dysfunction death (doubling of serum creatinine or eGFR < 50% from baseline)

^b^Renal recovery; serum creatinine level had returned to baseline or < 1.5 times from baseline and not ongoing need for RRT or currently receiving RRT (only patients who were non-recovery at discharge)

^c^New RRT; new case of RRT

^d^New CKD; newly diagnosis of chronic kidney disease by eGFR criteria in no previous history of CKD

^e^CKD progression; change of staging of CKD by eGFR criteria in previous history of chronic kidney disease

^f^Progression of albuminuria; change in staging of albuminuria criteria from at 3 months to 12 months post-discharge

^g^Recurrent AKI; document of AKI definition by KDIGO 2012 criteria

^h^Readmission; episode of unplanned readmission

^i^Blood pressure control; blood pressure < 140/90 mmHg in non-hypertensive patients or < 130/80 mmHg in hypertensive patients

^j^EQ-5D-5L index scores; descriptive system for health-related quality of life states in adults, consisting of five dimensions (Mobility, self-care, usual activities, pain and discomfort and anxiety and depression)

There was no meaningful difference in the reduction of serum creatinine between both groups (Additional file 1: Table S2, Figure S2). The absolute median change of eGFR from baseline to 12 months was not significant between both groups (Additional file 1: Table S3). The incidence of MAKE at 365 days, new RRT, new CKD, CKD progression, and renal recovery were comparable in both groups (*p* = 0.73, 0.62, 0.69, 0.41, 0.70 respectively) (Table 3).

### Non-renal outcomes

There was a significantly better blood pressure control in the comprehensive care group (87.9% vs. 57.6%; *p* = 0.006) (Table 3). However, the rate of RAASI use was not significantly different between the two groups (54.5% vs. 42.4%; *p* = 0.33), nor was the readmission rate (55.1% vs. 49%; *p* = 0.54). The QOL (EQ-5D-5L index score) tended to be better in the comprehensive care group, but this was not significant (0.99 [0.8–1.0] vs. 0.96 [0.8–1.0], *p* = 0.80). The nutrition, as shown by the 24-h urine sodium and protein intakes, was not significantly different between the two groups (112.82 ± 63.17 vs. 127.69 ± 54.23 mmol, *p* = 0.35; and 1.01 ± 0.32 vs. 0.85 ± 0.28 g/kg/d, respectively). Other results between the two groups were not significantly different (Additional file 1: Table S5). Interestingly, only 27.3% (nine patients) in the standard care group received nephrology consultation.

## Discussion

Our study was the first RCT to explore the outcomes of comprehensive care by a MDCT with post-severe AKI patients. We demonstrated the optimal process of care for post-severe AKI survivors according to the ADQI 16 Workgroup [12] and Quality Improvement Goals for AKI [28] guidelines. Our study showed the successful feasibility of a MDCT in post-AKI care, with significantly better rates of drug reconciliation, drug alert, and dietary recall.

Previously, post-AKI survivors were rarely followed up after discharge even though there have been many studies demonstrating impressive outcomes in patients receiving post-discharge care in other diseases, such as heart failure and myocardial infarction [29, 30]. A previous study showed that only 8.5% of AKI survivors had nephrology referrals after discharge [31], which is consistent with another retrospective study that showed that only 12% of post-AKI patients received a specialist nephrology follow-up, while only 57% had their serum creatinine level measured within 3–6 months following discharge [32]. Our study showed completely serum creatinine measurement in every visit in both comprehensive care group and standard care group, except those with loss to follow-up and death. We also showed successful post-AKI survivors follow-up by a nephrologist (100% in a comprehensive care group and 27.3% in standard care group, *p* < 0.001). (Table 2).

Moreover, post-AKI survivors had several co-morbid sequelae and may be taking many medications. These increase the risk of drug duplications, dosing error, drug interactions, or nephrotoxic drug prescription. In the comprehensive care group, we demonstrated the pharmacists’ role in drug records, drug dosing adjustment, and drug alerts to prevent such problems that may otherwise have harmed the patients. Our study showed a significant improvement in drug reconciliation and drug alert (100% vs. 0% and 33.3% vs. 0%, respectively, *p* < 0.001). As well as CKD patients, post-AKI survivors tend to have malnutrition that is related to their QOL and renal outcomes [33–35]. Nutritionists played a vital role in post-AKI follow-up as they provided food information, a guideline on the number of calories, minerals, and other nutrients by recording a 3-d dietary recall. A 3-d dietary recall is one of the most widely used tools in nutrition epidemiology to identify food, energy, and nutrient intake to evaluate diet assessment [36]. The 3-d dietary recall rate was significantly better in the comprehensive than in the standard care group (100% vs. 0%, respectively, *p* < 0.001) (Table 2).

The MDCT’s effect on the comprehensive care group resulted in the numerical reduction of the UACR (36.83 mg/g) compared to the standard care group (vs. 177.70 mg/g), and this was significantly lower at 6- and 12-month follow up after discharge (*p* = 0.044 and 0.036, respectively), as seen in Additional file 1: Table S4 and Fig. 2. The result may be from the optimized blood pressure control in the comprehensive care group more than the effect of RAASI, due to the comparable rate of RAASI usage in both groups. A recent study showed that a higher UACR was associated with a higher risk of CKD progression [37]. Our study did not demonstrate a significant lower rate of CKD progression in the comprehensive care group compared to the standard care group (63.6% vs 78.6%, *p* = 0.41). Despite a reduced UACR, the comprehensive care did not show any significant reduction in the MAKE at 365 d, new RRT, and renal recovery at 12 months, compared to the standard care (Table 3). However, our findings corresponded to a recent trial (FUSION trial) [38],which showed comparable MAKE365 outcome between post-AKI survivors follow-up by nephrologist versus usual care, (44% vs 43%, RR = 1.02, 95% CI 0.6–1.73).

We also reported the QOL in post-AKI survivors. The comprehensive care group tended to have a better QOL than the standard care group. This is an essential issue since severe AKI survivors had a lower physical and mental status than the normal population [39], so the improved QOL in these subgroups of patients could contribute to a better outcome.

Our study has several strengths. Firstly, we conducted the first RCT of implementing a MDCT (consisted of nephrologists, renal pharmacists, renal nutritionists, and nurses) in post-severe AKI survivors, especially in the high risk group (AKI requiring dialysis [AKI-D] and dialysis dependence at discharge) which were high risk for ESRD and mortality. Secondly, our MDCT completed all aspected of post-AKI health care bundles, being comprised of KAMPS (Kidney function, Advocacy, Medications, Pressure, and Sick day protocol) for all patients with AKI and WATCH-ME (Weight assessment, Access, Teaching, Clearance, Hypotension, and Medications) for AKI-D patients as recently proposed in the Quality Improvement Goal for AKI [28]. Thirdly, our study had the same format document and laboratory protocol in both comprehensive care group and standard care group, minimizing the ascertain bias and missing data problem. Finally, our study showed impressive outcomes of comprehensive care to reduce albuminuria, which is the hallmark of CKD progression.

Nevertheless, our study had several limitations. Firstly, the comprehensive care by the MDCT may not be available for centers in resource-limited settings. However, our study demonstated the process of care for AKI survivors; which may be aplied to other available clinics such as chronic kidney disease clinics (CKD clinics) or other metabolic clinics. Those clinics usually are available in resource-limited countries. Secondly, our patients had more severe AKI and higher rates of co-morbid diseases including diabetes, hypertension and CKD (87.8% of patients had stage 3 AKI, of which 76.5% were admitted to the ICU and 71.4% received RRT). Our results may not be generalizable to patients with other scenarios such as less severe AKI or non-ICU patients. Moreover, with the high number of patients on dialysis at discharge, this might affect the generalizability of the results. In many countries, these patients receive post AKI care from both nephrologist and multidisciplinary care as standard of care. Thirdly, our process of care which included the rate of medication reconciliation, rate of drug alert, and rate of 3-d dietary recall, might have favored the intervention group by its definition. However, we chose these parameters to reflect the frequently abandoned processes of care in real world practice. Of note, it is worth to mention that some of these actions might not have been captured due to the study definitions of MDC team and the limitation knowledge of internists. Fourthly, our study included patients with dialysis dependence at discharge (even though they would be followed up by nephrologists) because these patients were at high risk of renal non-recovery, new CKD, CKD progression, ESRD, and mortality. Correspondingly, the aim of our study was to explore the role of the multidisciplinary care team (nephrologists, renal pharmacists, renal nutritionists, and well-trained research co-ordinator nurses) in improving outcomes in these high-risk patients. Fifthly, our study lack of an intention to treat analysis and analyzed only patients who completed the study at 12 months. Finally, our study was only a pilot and feasibility study, and so the renal outcomes for implementation of comprehensive care may not be seen due to a type 2 error from the small sample size. Therefore, a larger population, extension of the follow-up time periods, and cost-effectiveness analyses should be assessed in future studies. The results of this feasibility trial showed that 32.5% in the comprehensive care group and 28.9% in the standard care group had MAKE365. Therefore, to detect a 10.0% difference in the MAKE between the comprehensive and standard care groups at a power of 80% and a 5% significance level, the minimum sample size to show the benefit of the MDCT (reduction of MAKE) approach over the standard should be at least 312 patients for each group.

## Conclusions

Our study explored the role of comprehensive care in improving the outcomes in post-severe AKI survivors. This study showed that comprehensive care by a MDCT is feasible and can be implemented for post-severe AKI survivors. Moreover, the comprehensive care group had better results in the reduction of UACR and blood pressure control. Further study with a larger number of patients should be conducted to establish the benefit of a MDCT approach in this high risk group.

## Supplementary Information

**Additional file 1**. **Table S1:** Responsibilities of the Multidisciplinary Care Team in Comprehensive Care. **Table S2:** Trends in the serum creatinine concentration and eGFR (mL/min/1.73 m^2^). **Table S3:** Absolute median change of estimated GFR (ml/min/1.73 m^2^) from baseline to 12 months follow-up. **Table S4:** Trend in the UACR. **Table S5:** Laboratory results at 12 months. **Figure S1:** Post AKI follow up form. **Figure S2:** Trend in the median serum creatinine concentration.

## Data Availability

On reasonable request, data from this study are available from the corresponding author.

## Abbreviations

ADQI: Acute disease quality initiative
AKI: Acute kidney injury
BPM: Blood pressure measurement
CKD: Chronic kidney disease
eGFR: Estimated glomerular filtration rate
EMR: Electronic medical record
KCMH: King Chulalongkorn Memorial Hospital
KDIGO: Kidney disease improving global outcomes
ICU: Intensive care unit
IQR: Interquartile range
MAKE: Major adverse kidney events
MDCT: Multidisciplinary care team
NGAL: Neutrophil gelatinase-associated lipocalin
QOL: Quality of life
RAASI: Renin-angiotensin-aldosterone-system inhibitor
RRT: Renal replacement therapy
SD: Standard deviation
UACR: Urine albumin creatinine ratio
