# Supplementary material for: Comprehensive versus standard care in post-severe acute kidney injury survivors, a randomized controlled trial
Source: Crit Care. 2021 Aug 31;25:322. doi: 10.1186/s13054-021-03747-7 (PMC8406590; doi:10.1186/s13054-021-03747-7)
Supplement: Supplementary file 1 — Additional file 1. Table S1: Responsibilities of the Multidisciplinary Care Team in Comprehensive Care. Table S2: Trends in the serum creatinine concentration and eGFR (mL/min/1.73 m2). Table S3: Absolute median change of estimated GFR (ml/min/1.73 m2) from baseline to 12 months follow-up. Table S4: Trend in the UACR. Table S5: Laboratory results at 12 months. Figure S1: Post AKI follow up form. Figure S2: Trend in the median serum creatinine concentration. [file 13054_2021_3747_MOESM1_ESM.docx]

**Table S1: Responsibilities of the Multidisciplinary Care Team in Comprehensive Care**

| **Nephrologists** |
| --- |
| - Co-ordination with other MDCT members - Management of post-AKI transitional care: renal recovery evaluation, prevention of CKD progression, preparation of RRT modality - Management of dialysis related conditions: dialysis prescription, vascular access preparation, dry weight adjustment - Management of medical related conditions: blood pressure control, blood sugar control, anemia management, bone and mineral disease management, volume management, cardiovascular risk management |
| **Renal Pharmacists** |
| - Drug reconciliation - Drug alert: notification of medication that potential harmful or conflict or discrepancy of medication - Medication dosage adjustment based on renal function - Medication advice: NSAID avoidance, nephrotoxin avoidance, sick day protocol - Pre-operative medication management |
| **Renal Nutritionists** |
| - Dietary and nutritional counseling - Nutritional assessment - Dietary compliance by 3-d dietary recall |
| **Nurses** |
| - Coordinator between the patients and the MDCT: patients’ appointments, encourage follow-up by phone and LINE application - Laboratory test and blood pressure monitoring - Quality of life measurement using the EQ-5D-5L index scores |

**
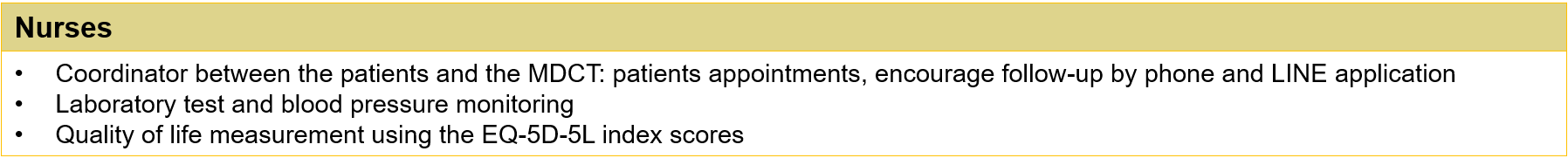

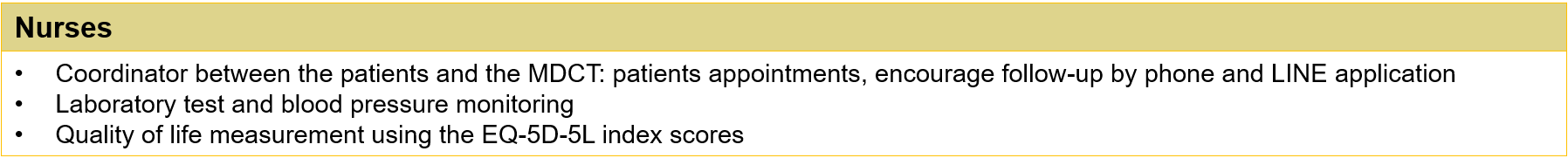

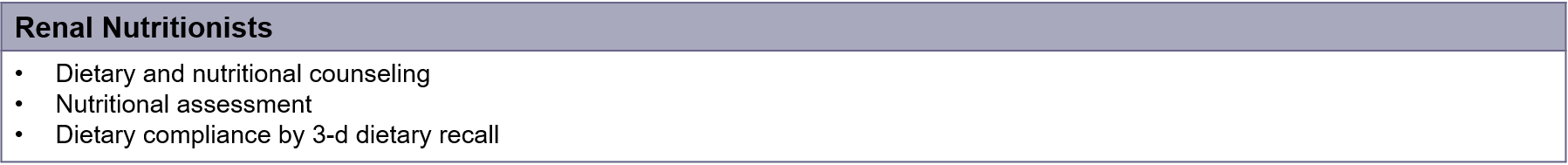
**

**Table S2: Trends in the serum creatinine concentration and eGFR (mL/min/1.73 m^2^)**

|  | Comprehensive care | | Standard care | | P-value^a^ | P-value^b^ |
| --- | --- | --- | --- | --- | --- | --- |
|  | Creatinine  (mg/dL) | eGFR  (mL/min/1.73 m^2^) | Creatinine  (mg/dL) | eGFR  (mL/min/1.73 m^2^) |  |  |
| At discharge | 1.53 (1.04, 2.93)  N = 41 | 42.14 (18.73, 71.90)  N = 41 | 1.71 (1.29, 2.49)  N = 41 | 32.06 (21.57, 56.70)  N = 41 | 0.66 | 0.84 |
| At 3 months | 1.19 (0.95, 1.93)  N = 41 | 51.14 (32.54, 83.62)  N = 41 | 1.37 (0.86, 2.24)  N = 44 | 45.60 (26.30, 77.20)  N = 44 | 0.56 | 0.51 |
| At 6 months | 1.31 (0.98, 1.90)  N = 34 | 54.24 (31.05)  N = 34 | 1.31 (0.88, 2.03)  N = 36 | 54.99 (30.38)  N = 36 | 0.96 | 0.92 |
| At 9 months | 1.14 (0.91, 1.56)  N = 29 | 61.36 (32.46)  N = 29 | 1.18 (0.92, 1.90)  N = 31 | 55.80 (30.31)  N = 31 | 0.60 | 0.50 |
| At 12 months | 1.14(0.80,1.46)  N = 27 | 66.74(30.77)  N = 27 | 1.05(0.84,2.08)  N = 29 | 61.23(35.16)  N = 29 | 0.49 | 0.54 |

Data excluded patients with RRT and are shown as median (IQR) or mean (SD).

^a,b^ p-value comparison of the ^a^serum creatinine and ^b^eGFR between the standard and comprehensive care groups.

**Table S3:** Absolute median change of estimated GFR (ml/min/1.73 m^2^) from baseline to 12 months follow-up

| Parameters | Comprehensive care | Standard care | P-value |
| --- | --- | --- | --- |
| All patients | 3.90(-3.57,13.06)  N=33 | 9.35(-3.73,27.82)  N=33 | 0.57 |
| Patients without CKD | 4.01(-7.60,23.80)  N=22 | -0.59(-17.72,29.31)  N=19 | 0.83 |
| Patients with CKD | 3.90(-1.42,7.46)  N=11 | 10.17(-0.31,25.96)  N=14 | 0.11 |
| Patients with new CKD | 38.41(33.35,-)  N=3 | 35.35(27.94,72.38)  N=4 | 0.72 |
| Patients with CKD progression | 7.26(2.72,7.46)  N=7 | 10.75(0,25.29)  N=11 | 0.09 |

Data are excluded patients with lost to follow-up and death at 12 months

Data are presented as median (IQR)

**Table S4: Trend in the UACR**

|  | Comprehensive care |  | Standard care |  | P-value |
| --- | --- | --- | --- | --- | --- |
| At 3 months^a^ | 78.07 (11.15, 210.55)  N = 33 |  | 115 (35.12, 464.60)  N = 37 |  | 0.15 |
| At 6 months^b^ | 36.50 (9.51, 227.95)  N = 30 |  | 154.14 (18.65, 613.27)  N = 34 |  | **0.044** |
| At 9 months^c^ | 29.34 (7.59, 138.55)  N = 27 |  | 113.07 (7.50,550.99)  N = 30 |  | 0.29 |
| At 12 months^d^ | 36.83 (13.39,131.90)  N = 24 |  | 177.70 (47.12,745.71)  N = 26 |  | **0.036** |

Data excluded patients with RRT and missing data, and are shown as the median (IQR).

^a^ Missing data; comprehensive care (n=8), standard care (n=7)

^b^ Missing data; comprehensive care (n=4), standard care (n=2)

^c^ Missing data; comprehensive care (n=2), standard care (n=1)

^d^ Missing data; comprehensive care (n=3), standard care (n=4)

**Table S5: Laboratory results at 12 months**

| Parameters | Comprehensive care | Standard care | P-value |
| --- | --- | --- | --- |
| HbA1C (%) | 5.90 (5.25, 7.05) | 5.90 (5.23, 7.05) | 0.91 |
| LDL (mg/dL) | 100.94 (38.11) | 84.19 (36.52) | 0.08 |
| Hb (g/dL) | 12 (10.4, 13.75) | 11.9 (9.7, 13.45) | 0.72 |
| Potassium (mmol/L) | 4.02 (0.50) | 3.99 (0.51) | 0.83 |
| Bicarbonate (mmol/L) | 26.52 (2.58) | 26.12 (3.40) | 0.59 |
| Calcium (mg/dL) | 9.38 (0.56) | 9.35 (0.71) | 0.85 |
| Phosphate (mg/dL) | 3.47 (0.66) | 3.57 (0.67) | 0.55 |

Data are shown as the mean (SD) or median (IQR)

**Figure S1: Post AKI follow up form**

**
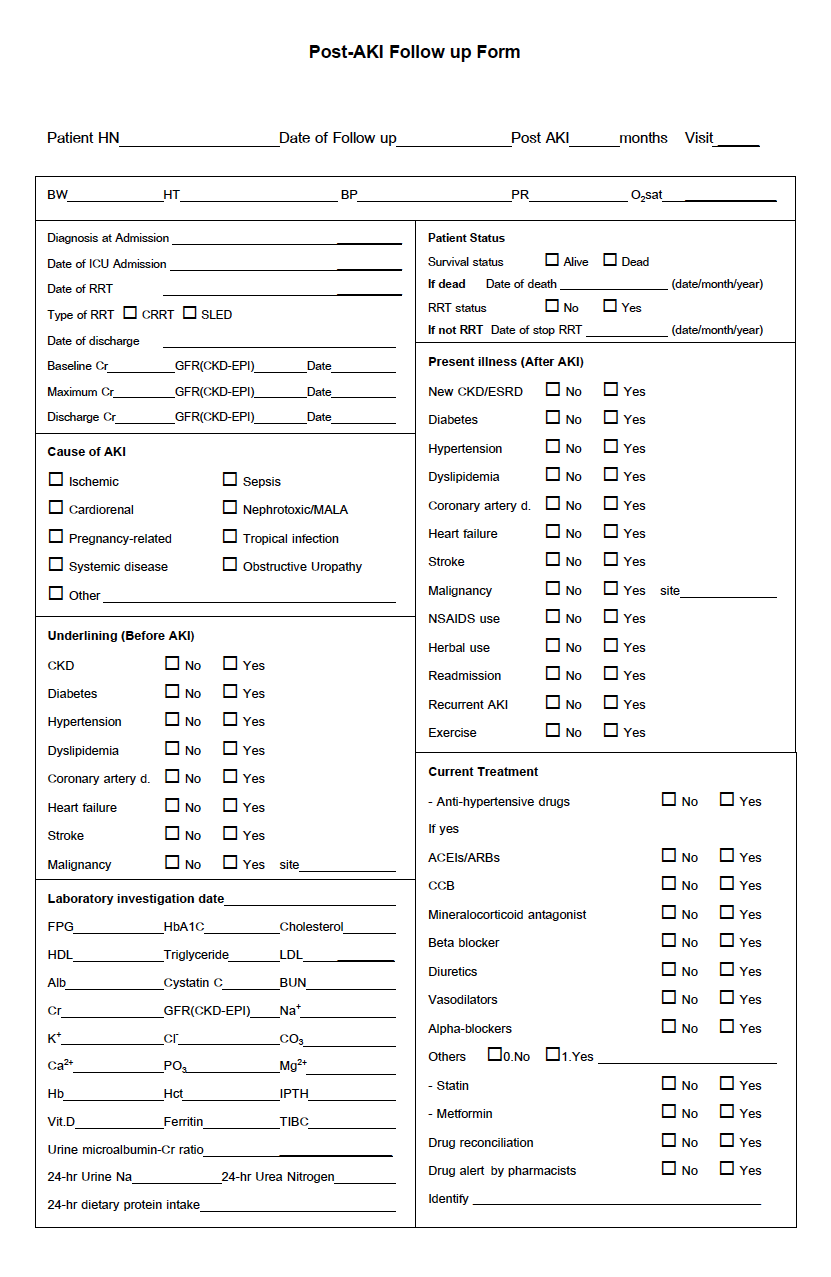
**

**Figure S2: Trend in the median serum creatinine concentration**

Data are shown as the median (IQR) and patients with RRT or missing data were excluded.
